# Potentially Inappropriate Prescribing of Oral Solid Medications in Elderly Dysphagic Patients

**DOI:** 10.3390/pharmaceutics10040280

**Published:** 2018-12-16

**Authors:** Matteo Sestili, Serena Logrippo, Marco Cespi, Giulia Bonacucina, Letizia Ferrara, Silvia Busco, Iolanda Grappasonni, Giovanni Filippo Palmieri, Roberta Ganzetti, Paolo Blasi

**Affiliations:** 1 Hospital Pharmacy, Italian National Research Center on Aging (INRCA), via della Montagnola 81, 60127 Ancona, Italy; matteo.sestili@sanita.marche.it; 2 School of Pharmacy, University of Camerino, via Gentile III da Varano, 62032 Camerino, Italy; serena.logrippo@unicam.it (S.L.); marco.cespi@unicam.it (M.C.); giulia.bonacucina@unicam.it (G.B.); silvia.busco@studenti.unicam.it (S.B.); iolanda.grappasonni@unicam.it (I.G.); gianfilippo.palmieri@unicam.it (G.F.P.); paolo.blasi@unicam.it (P.B.); 3 International School of Advanced Studies (ISAS), University of Camerino, Via Camillo Lili 55, 62032 Camerino, Italy; 4 Medical Direction, Italian National Research Center on Aging (INRCA), via della Montagnola 81, 60127 Ancona, Italy; l.ferrara@inrca.it

**Keywords:** old-old patients, inappropriate prescriptions, solid oral dosage form, tablets, capsules, compounding, manipulation

## Abstract

Pharmaceutical formulations suitable for dysphagic patients are not always commercially available, motivating caregivers to crush tablets or open capsules to facilitate swallowing. Since this action may modify the characteristics of the medicine, it should be considered potentially inappropriate. This paper is the first to focus on how hospitalization affected the rate of potentially inappropriate prescriptions (PIPs) and the incidence of dosage form-related PIPs in elderly patients with dysphagia. Data was collected by reviewing patient medical records in the Italian National Research Center on Aging of Ancona. The therapy at admission and discharge was analysed in terms of: inappropriate drug associations, inappropriate drugs for dysphagic patients, inappropriate dosage forms and inappropriate dosage form modifications. Forty-one dysphagic patients with an average age of 88.3 years were included in the study and 451 prescriptions were analysed. PIPs were widespread at admission, and hospitalization did not improve the situation in a statistically significant manner. The most common PIPs identified (>80%) were related to dosage form selection and modification. This study highlights a clear need for continuing medical education about prescription appropriateness and modification of solid dosage forms in patients with dysphagia.

## 1. Introduction

Dysphagia (also called oropharyngeal dysphagia), defined as a condition involving perceived or real difficulty in forming or moving a bolus safely from the oral cavity to the oesophagus, is a common issue in the elderly population [1,2]. Although the exact prevalence of dysphagia is difficult to determine, it is estimated to affect around 55% of patients in aged care settings [3] and is expected to increase in the near future due to the increasing proportion of the elderly population [4]. 

Dysphagia in the elderly is associated with physiologic deficits in the mouth, pharynx, larynx and oesophagus, connected to aging, as well as to events and conditions affecting the central nervous system [5,6,7] and to drug treatment [8,9]. Dysphagia affects not only the ingestion of food but also that of solid oral dosage forms (SODFs), the most prescribed dosage forms, with complications that include poor adherence to therapy and even treatment failure [10,11,12,13,14]. The scenario is complicated by the fact that older adults, the population most predisposed to develop dysphagia, often have comorbidities and take a number of medicines, conditions that increase the frequency of medication errors [15,16,17].

In patients with dysphagia (PWD), the prescription of SODFs should be avoided as much as possible. Physicians should opt for an appropriate formulation, such as a syrup, when it is available on the market, or an alternative route of administration. When this is not the case, physicians are obliged to prescribe SODFs, such as tablets or capsules, which must be modified by crushing or opening them to allow for easier swallowing [18]. The practice of crushing tablets or opening capsules is very common in geriatric wards and nursing homes, but its consequences are often overlooked [19,20,21]. These actions can modify the pharmacokinetics of the medicinal product by compromising its established bioavailability, effectiveness and toxicity [14,22,23]. In fact, guidelines on the precautions to be used during medicinal product modification are now available [24,25]. For instance, extended-release formulations, such as prolonged-release or enteric-coated forms, should not be crushed in order to avoid adverse events due to changes in pharmacokinetics [25,26].

The prescription of SODFs to PWD when a more adequate formulation is available on the market is effectively an inappropriate prescription or at least a potentially inappropriate prescription (PIP) that can be numbered among medication errors. Medication errors have been defined as “any preventable event that may cause or lead to inappropriate medication use or patient harm while the medication is in the control of the health care professional, patient, or consumer” [27]. These errors can happen at any stage of medication management and they are usually classified in the categories of mistakes in: ordering/prescribing, transcribing and verifying, dispensing and delivering, administering, monitoring and reporting errors [28]. 

Among the various classes of medication errors, medicine administration errors (MAEs), defined as “deviation from the prescriber’s order as written on the patient’s drug chart” [29], have been widely investigated in the elderly. In the specific case of elderly PWD, the most recent studies highlight a higher MAE frequency in PWD compared to that in the control group [15,30]. Similar findings were reported by Haw et al. [31], by comparing MAE frequency in psychiatric inpatients with and without swallowing difficulties. 

Inappropriate prescription is a type of medication error with a high frequency in the elderly population. A review of 11 studies published from 1997 to 2001 showed a PIP rate ranging from 21% to 40%, as a function of polypharmacy, health status and gender [32]. A more recent collection of studies on PIPs in the elderly reported comparable values, even though large differences were recorded as a function of the nation considered [33]. Although these studies used different criteria to detect PIPs, all can be considered as potentially inappropriate prescribed medicines. The high incidence of PIPs in elderly PWD is common knowledge, but few reports have investigated their incidence in elderly with and without dysphagia [30]. 

In the fall of 2014, a multidisciplinary team (doctors, nurses, pharmacists, speech and language therapists) of the Italian National Research Center on Aging (INRCA) geriatric hospital in Ancona, Italy wrote medical review guidelines for PWD and distributed them to its healthcare professionals to help them assess the medication regimen of these patients in order to avoid PIPs.

The aim of this study was to evaluate the effect of hospitalization on the rate of PIPs in elderly PWD through a retrospective analysis of the medication lists at admission and discharge in the INRCA geriatric hospital. Our study focused on the third category identified by Beers: “medications that should not be used in persons known to have specific medical conditions, even though their use in the general population of elders might be appropriate” [34]. Emphasis was given to active pharmaceutical ingredients (APIs) and dosage forms that should not be used in dysphagic patients. PIPs were subdivided into: inappropriate drug association (IDA), inappropriate drug for dysphagic patient (IDDP), inappropriate dosage form for dysphagic patient (IDF) and inappropriate dosage form modification (IDM).

## 2. Materials and Methods

### 2.1. Sample and Data Collection

The medication lists at admission and the discharge letters (attached to the medical records) of patients hospitalized between January and April 2015 in the INRCA (Ancona) in two elderly care units (geriatric unit 1 and 2) and one neurological unit were analysed. All the patients involved in the study were selected according to the following criteria:

- Age >79 years (categorized as old-old [35]);
- Diagnosis of dysphagia carried out by the speech and language therapists before hospital admission or during hospitalization. No patients involved in this study were able to swallow SODFs.

Data collection was carried out through the review of medical records in collaboration with caregivers and the medical and nursing staff of the wards involved.

Age, gender, previous hospitalizations during the last year, the name of the inpatient ward, any suspicion of aspiration pneumonia at admission and the drugs prescribed before (admission) and after the hospitalization (discharge) were recorded for all the patients.

PIPs were grouped according to the following criteria:

- IDA: simultaneous prescription of APIs that should not be associated, according to the Lexicomp^®^ database (x grade) [36];
- IDDP: prescription of a drug which can induce dysphagia (drug-induced dysphagia) identified according to the following criteria [8,9]:
  ▪ Dysphagia is a side effect of the drug;
  ▪ Dysphagia is a complication of the drug’s therapeutic action;
  ▪ Medication-induced oesophageal injury.

Only drugs with these side effects reported as “common” (≥1/100 and <1/10) or “very common” (≥1/10) were considered. Examples of drugs that can induce dysphagia are substances with anticholinergic or antimuscarinic activity, or those associated with dry mouth (xerostomia) [7,9,37,38].

- IDF: prescription of a medicine having an inappropriate dosage form for PWD (tablets or capsules) when more suitable formulations or alternative routes of administrations are commercially available (oral solutions or trans-dermal patches) [39,40];
- IDM: SODFs that should not be manipulated, such as enteric-coated tablets or extended release formulations [25,41,42].

### 2.2. Sample Size

Assuming a 30% incidence of the PIP rate in the elderly population [32,33], 323 prescriptions and 144 prescriptions were required to obtain a 95% interval of confidence (CI) of ±5% and ±7.5%, respectively. Given that Italians between the ages of 80 and 84 years take an average of 7.4 prescriptions [43], 44 patients and 19 patients represent adequate sample sizes to obtain the 95% CI of ±5% and ±7.5%.

### 2.3. Data Analysis

All the collected data were summarized using descriptive statistics. Hypothesis testing was performed to analyse the effects of hospitalization. The number of prescriptions for each patient and the number of inappropriate prescriptions for each patient at admission (*n* = 29) and discharge (*n* = 29) were compared using the paired t-test (significance level *p* < 0.05; normality of mean difference data evaluated using the Anderson–Darling test). The proportions of the inappropriate and appropriate prescriptions at admission (*n* = 41) and discharge (*n* = 29) were compared using the Fisher’s Exact test (significance level *p* < 0.05). Data are reported as mean ± standard deviation unless otherwise specified.

## 3. Results

The general characteristics of the population are summarized in Table 1. From admission to discharge, the number of patients considered in this study decreased by 12 (29%), due to exitus or transfer to other wards. A diagnosis of dysphagia was present at admission in 92% of the patients, while at discharge this percentage reached 100%. Around 27% of the patients had enteral feeding when admitted. This percentage increased to around 28% when the patients were discharged, but this variation was exclusively related to the reduction of the number of patients considered in the study. In fact, only 29 of the 41 patients had a discharge letter, while 12 did not, due to exitus (*n* = 7) or transfer to another hospital ward (*n* = 5). Thus, of the 41 patients included in the study, 11 (27%) had enteral feeding tubes at admission: considering just the 29 with a discharge letter, the number was eight patients (28%), as seen in Table 1.

**Figure 1:**
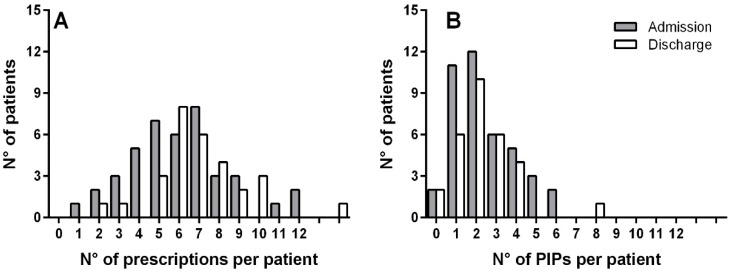
Distribution of (**A**) number of prescriptions per patient and (**B**) number of potentially inappropriate prescriptions (PIPs) per patient at admission and discharge.

The hospitalization (mean length 15 days) determined a slight increase of the total prescriptions (TPs) of around 6%. The mean TPs for patients at admission and at discharge were not statistically different (95% CI for the mean differences −0.3–1.6) when tested with the paired t-test on the 29 patients admitted and discharged with discharge letters. The distribution of the number of prescriptions at admission and discharge is reported in Figure 1A. 

The presence of PIPs was widespread; only 4.9% of the patients at admission (*n* = 41) showed a medication list without PIPs, and the mean number of PIPs per patient was 2.4 ± 1.5. At discharge (*n* = 29), the percentage of patients without PIPs rose to 6.9%, while the mean PIPs per patient remained similar (2.3 ± 1.6). A summary of the number and mean values of appropriate and PIPs at admission and discharge is reported in Table 2.

The mean PIPs for patient at admission and discharge (taking into account only the 29 patients discharged) was not statistically different (95% CI for the mean differences −0.6–0.8, paired t-test). The same results were found when comparing the proportions of appropriate and PIPs (*p*-value = 0.142, Fisher’s Exact test) between the two groups (*n* = 41 versus *n* = 29). The distribution of the number of PIPs at admission and discharge is reported in Figure 1B.

**Figure 2:**
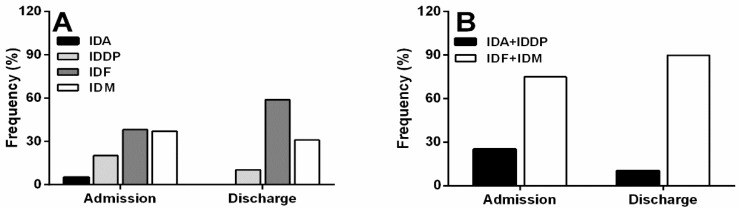
Percentage distribution of (**A**) the four different PIPs at admission and discharge and (**B**) the PIPs grouped as API-related PIPs (IDA + IDDP) and dosage form-related PIPs (IDF + IDM) at admission and discharge. Abbreviations: PIPs, potentially inappropriate prescriptions, API, active pharmaceutical ingredient; IDA, inappropriate drug association; IDDP, inappropriate drug for dysphagic patient; IDF, inappropriate dosage form for dysphagic patient; and IDM, inappropriate dosage form modification.

The frequency percentage of the different types of PIPs at admission and discharge is reported in Figure 2A. For the 29 patients admitted and discharged, the most diffused PIP was IDF (around 48% of all the PIPs, by counting together PIPs at admission and discharge), followed by IDM (32%), IDDP (17%) and IDA (4%). The most common IDF was associated with the diuretic furosemide (around 40%), prescribed as tablets, while a liquid oral formulation was available. The compounding of gastro-resistant tablets containing acetylsalicylic acid, pantoprazole and lansoprazole was the most common IDM, followed by the modification of extended release oral dosage forms of trazodone and tamsulosine. Quetiapine and olanzapine were the most common IDDPs prescribed [9,44], while the most frequent IDA was the association of esomeprazole with clopidogrel (bearing in mind that only five IDAs were detected). All the prescriptions considered potentially inappropriate, together with the reasons, are reported in supplementary Table S1a–d.

For a better understanding, data of prescriptions at admission (*n* = 41) and discharge (*n* = 29) were grouped into PIPs that are API-related (IDA + IDDP) and dosage form-related (IDF + IDM). The frequency percentage at admission and discharge of each group of PIPs is reported in Figure 2B. Comparing the proportions of the grouped PIPs, statistically significant differences were observed (*p*-value = 0.018, Fisher’s Exact test). Analysis of the adjusted standardised residues [45,46] indicates that both the increase of the PIPs related to the dosage forms and the decrease of the PIPs related to drug were wider than would be expected based on chance alone. 

## 4. Discussion

Beginning in the 1990s, different criteria for identifying and quantifying PIPs in the general elderly population have been defined [34,47,48,49,50,51,52,53] and guidelines for measuring and avoiding this problem have been implemented. However, these studies and guidelines have not specifically included elderly dysphagic patients. The present study evaluates for the first time the rate of PIPs in elderly PWD by considering not only the appropriateness of the APIs prescribed, but also the suitability of the dosage form. The major role of PIPs related to dosage form is evident from the analysis of the collected data. As seen in Table 2, most of the prescriptions were considered potentially inappropriate because of dosage form selection and modification. Dosage form-related PIPs were more frequent compared to API-related PIPs. 

Interesting results come from the effect of hospitalization on prescription appropriateness. Hospitalization was responsible for a non-statistically significant reduction of the number of patients without PIPs as well as a decrease of the total PIPs. However, by using Fisher’s Exact test to analyse the variation of proportions of the clustered PIPs, statistically significant changes between admission and discharge emerged. At discharge, API-related PIPs decreased while dosage form-related PIPs increased. The analysis of the adjusted standardized residues indicated that both changes were wider than those that would be expected based on chance alone. Therefore, the errors related to dosage forms persisted and even increased with hospitalization.

It is important to highlight that this work is a retrospective study and the API-related PIPs can be considered as such only from a general point of view. In fact, it is likely that the physicians evaluated every single case and decided the prescriptions according to risk/benefit criteria, though the same consideration may not be valid for the PIPs related to the dosage forms. Another obvious limitation of the study is the impossibility of analysing to what extent clinical problems occurred as a consequence of the PIPs identified. 

Since the problem is not new, different clinical governing bodies and associations in Europe and the United States of America have suggested the use of a collaborative, multidisciplinary team approach for the management of dysphagia. For instance, the British Geriatrics Society issued a Best Practice Guide entitled “Dysphagia Management for Older People Towards the End of Life”, recommending, among others “[a] holistic patient-centred multidisciplinary team approach to dysphagia management” [54]. Recently, the Scottish Intercollegiate Guidelines Network (SIGN) also issued a national clinical guideline entitled “Management of Patients with Stroke: Identification and Management of Dysphagia”, which encouraged caregivers to explore the availability of alternative formulations or routes of administration, since crushing tablets to allow easier swallowing is not always appropriate. The SIGN guidelines also stated: “Hospital and community pharmacists or medicines information centres should be consulted by the professional managing the patient’s dysphagia, on the most appropriate method of administering medication” [55].

It is evident that the scientific community is moving toward the correct management of pharmacological therapy in PWD. However, the present study, while performed on a limited number of patients, nonetheless underlines the need for greater awareness when prescribing SODFs to dysphagic patients. Special attention must be paid to extended-release formulations because unsuitable modifications can seriously compromise the pharmacokinetics and/or the pharmacodynamics of the medicinal product and lead to severe side effects. The introduction of a “do not crush” label [56,57,58] could be useful for improving the prescription and administration of oral therapies to dysphagic patients.

## 5. Conclusions

Choosing the appropriate oral therapy for patients with dysphagia is a special challenge. Previous studies have already highlighted the importance of close collaboration between doctors, nurses and pharmacists to reduce PIPs and avoid the administration of SODFs to PWD.

The results of this study show a high number of PIPs in elderly patients with dysphagia, mainly due to the selection of the wrong dosage forms or to the inadvisable modification (compounding) of medicines. According to our data, hospitalization determines only a slight reduction of the PIPs, which was not statistically significant. Due to the high frequency of reports of medication errors in the literature, the application of a multidisciplinary approach and the introduction of corrective measures to reduce the PIP rate should be mandatory.

## Figures and Tables

**Table 1:** General characteristics of the patients.

Patient Characteristics	Patients Enrolled in the Study	Patients not Discharged from the Ward *	Patients Discharged from the Ward
Number of patients	41	12	29
Geriatric 1 unit	17	4	13
Geriatric 2 unit	16	4	12
Neurological unit	8	4	4
Age (year), mean (SD)	88.3 (5.4)	87.9 (4.9)	88.5 (5.6)
Males, no (%)	19 (46)	6 (50)	13 (45)
Number of patients with dysphagia diagnosis before admission (%)	38 (92)	11 (92)	27 (93)
Number of patients with dysphagia diagnosis during hospitalization (%)	3 (8)	1 (8)	2 (7)
Number of patients with enteral feeding tubes at admission (%)	11 (27)	3 (25)	8 (28)
Nasogastric tube (%)	7 (17)	1 (8)	6 (21)
Percutaneous endoscopic Gastrostomy tube (%)	4 (10)	2 (17)	2 (7)
Number patients with suspected aspiration pneumonia at admission (%)	13 (32)	4 (33)	9 (45)
Number patients with previous hospitalizations during the last year (%)	25 (61)	4 (33)	21 (72)
Hospitalization (days), mean (SD)	-	-	15.2 (7.2)

* For 12 of the 41 patients enrolled in the study, there were no discharge letters, due to exitus (*n* = 7) or transfer to another hospital ward (*n* = 5).

**Table 2:** Number and mean values of appropriate and PIPs at admission and discharge.

Prescription Data	Admission		Discharge
	*n* = 41	*n* = 29 *	*n* = 29
Total number of prescriptions	247	185	204
Median number of prescriptions for patients (95% CI)	6 (5–7)	6 (5–7)	7 (6–8)
Total number of PIPs (%)	100 (41)	64 (35)	68 (33)
IDA (%)	5 (5)	5 (8)	0 (0)
IDDP (%)	20 (20)	15 (23)	7 (7)
IDF (%)	38 (38)	23 (36)	40 (59)
IDM (%)	37 (37)	21 (33)	21 (31)
Median number of PIPs for patients (95% CI)	2 (2–3)	2 (1–3)	2 (2–3)
Number of patients without PIPs (%)	2 (5)	2 (7)	2 (7)

* The column refers to the 29 patients admitted and then discharged (12 patients out of 41 were without discharge letter due to exitus or transfer to another hospital ward). Abbreviations: CI, confidence interval; IDA, inappropriate drug association; IDDP, inappropriate drug for dysphagic patient; IDF, inappropriate dosage form for dysphagic patient; and IDM, inappropriate dosage form modification.

## Supplementary Materials

The following are available online at http://www.mdpi.com/1999-4923/10/4/280/s1, Table S1a–d: A complete list of all the prescriptions considered inappropriate at admission and discharge.
