# Supplementary material for: Potentially Inappropriate Prescribing of Oral Solid Medications in Elderly Dysphagic Patients
Source: Pharmaceutics. 2018 Dec 16;10(4):280. doi: 10.3390/pharmaceutics10040280 (PMC6321461; doi:10.3390/pharmaceutics10040280)
Supplement: Supplementary file 1 [file pharmaceutics-10-00280-s001.pdf]

Supplementary Tables S1a-S1d

Table S1a: Complete list of all the prescriptions considered as inappropriate drug association (IDA) at admission and discharge. N° represents the number of patients having this prescription, API is the active pharmaceutical ingredient, DF is the dosage forms, and Note report the reason of inappropriateness.

Admission				Discharge			
N°	API	DF	Note	N°	API	DF	Note
1	Olanzapine-Alprazolam		X grade Lexicomp® database	No inappropriate drug association detected			
2	Clopidogrel-Esomeprazole		X grade Lexicomp® database				
2	Quetiapine-Tetrabenazine		X grade Lexicomp® database				

Table S1b: Complete list of all the prescriptions considered as inappropriate drug for dysphagic patients (IDDP) at admission and discharge. N° represents the number of patients having this prescription, API is the active pharmaceutical ingredient, DF is the dosage forms, and Note report the reason of inappropriateness.

Admission				Discharge			
N°	API	DF	Note*	N°	API	DF	Note*
5	Olanzapine	Orodispersible tablets	A + B + C + D	4	Quetiapine	Conventional tablets	A + B + C +D
4	Quetiapine	Conventional tablets	A +B + C + D	1	Fentanyl	Transdermal patch	A + D
2	Doxazosine	Conventional tablets	A	1	Glycopyrronium	Dry powder inhaler	A
1	Alprazolam	liquid dosage form	A + B	1	Risperidone	Liquid dosage form	A + B + C + D
1	Fentanyl	Transdermal patch	A + D				
1	Glycopyrronium	Dry powder inhaler	A				
1	Mirtazapine	Orodispersible tablets	A + B + D				
1	Paroxetine	Liquid dosage form	A + B				
1	Perindopril and Indapamide	Conventional tablets	A				
1	Risperidone	Liquid dosage form	A + B + C + D				
1	Donperidone	Liquid dosage form	A + C				
1	Clonidine	Transdermal patch	A				

* A = Dry mouth.
B = Detrimental effects on consciousness or oropharyngeal swallow response.
C = Hyperfunctional involuntary movements (dyskinesia) of the oral pharyngeal muscolature, rigidity and spasm of the pharyngeal musculature.
D = Sedation.

Table S1c: Complete list of all the prescriptions considered as inappropriate dosage form (IDF) at admission and discharge. N° represents the number of patients having this prescription, API is the active pharmaceutical ingredient, DF is the dosage forms, and Note report the reason of inappropriateness.

Admission				Discharge			
N°	API	DF	Note	N°	API	DF	Note
16	Furosemide	Conventional tablets or Injection	Available as liquid oral dosage form.	14	Furosemide	Conventional tablets or Injection	Available as liquid dosage form.
3	Lansoprazole	Enteric tablets	Available as orodispersible tablets.	7	Lansoprazolo	Enteric tablets	Available as orodispersible tablets.
3	Levothyroxine	Conventional tablets	Available as liquid dosage form.	3	Losartan	Conventional tablets	Available as liquid dosage form.
2	Polyethylene Glycol 3350	Powder for water dispersion*	Available as liquid dosage form.	2	Ciprofloxacine	Conventional tablets	Available as liquid dosage form.
2	Valproate	Conventional tablets	Available as liquid dosage form.	2	Levodopa/carbidopa	Conventional tablets	Water dispersible tablets
1	Codeine and Acetaminophen	Conventional tablets	Available as granules for oral Suspension.	2	Levothyroxine	Conventional tablets	Available as liquid dosage form.
1	Domperidone	Conventional tablets	Available as liquid dosage form.	2	Polyethylene Glycol 3350	Powder for water dispersion*	Available as liquid dosage form.
1	Esomeprazole	Water dispersible tablets**	Available as water dispersible powder dosage form.	1	Esomeprazole	Water dispersible tablets**	Available as water dispersible powder dosage form.
1	Phenobarbital	Conventional tablets	Available as injection	1	Phenobarbital	Conventional tablets	Available as injection
1	Lormetazepam	Conventional tablets	Available as liquid dosage form.	1	Lormetazepam	Conventional tablets	Available as liquid dosage form.
1	Losartan	Conventional tablets	Available as liquid dosage form.	1	Mirtazapine	Conventional tablets	Available as orodispersible tablets.
1	Acetaminophen	Conventional tablets	Available as granules for oral Suspension and effervescent tablets.	1	Olanzapine	Conventional Tablets	Available as orodispersible tablets.
1	Digoxin	Conventional tablets	Available as liquid dosage form.	1	Acetaminophen	Injection	Available as granules for oral Suspension and effervescent tablets.

1	Promazine	Injection	Available as liquid dosage form.	1	Paroxetine	Conventional Tablets	Available as liquid dosage form.
1	Cefixime	Conventional tablets	Available as orodispersible tablets.	1	Trazodone	Conventional Tablets	Available as liquid dosage form.
1	Levodopa and Benserazide	Conventional tablets	Water dispersible tablets				
1	Torasemide	Conventional tablets	Available as injection				

* Polyethylene glycol 3350 dispersible powder is not indicated for PWD due the high water amount necessary for the reconstitution (> 100 mL). The concentrated liquid formulation is strongly suggested for PWD.

** In PWD requiring jellified vehicles, Nexium® granules is the most suitable formulation; while Nexium® tablets are appropriate for patients under enteral nutrition (see manufacturer information).

Table S1d: Complete list of all the prescriptions considered as inappropriate dosage form modification (IDM) at admission and discharge. N° represents the number of patients having this prescription, API is the active pharmaceutical ingredient, DF is the dosage forms, and Note report the reason of inappropriateness.

Admission				Discharge			
N°	API	DF	Note	N°	API	DF	Note
10	Pantoprazole	Enteric tablets	Unstable in the gastric environment.	5	Lansoprazole	Enteric tablets	Unstable in the gastric environment.
8	Acetylsalicylic acid	Enteric tablets	harmful for gastric mucosa.	4	Pantoprazole	Enteric tablets	Unstable in the gastric environment.
4	Lansoprazole	Enteric tablets	Unstable in the gastric environment	3	Tamsulosin	Extended release capsules	Drug overdose
4	Trazodone	Extended release tablets	Drug overdose	2	Acetylsalicylic acid	Enteric tablets	Unstable in the gastric environment.
3	Iron sulfate	Enteric tablets	harmful for gastric mucosa.	2	Oxycodone /Naloxone	Extended release tablets	Drug overdose
2	Tamsulosin	Extended release capsules	Drug overdose	1	Valproate	Extended release tablets	Drug overdose
2	Isosorbide	Extended release tablets	Drug overdose	1	Biperiden	Extended release tablets	Drug overdose
1	Nifedipine	Extended release tablets	Drug overdose	1	Dabigatran	Capsules	Drug overdose
1	Biperiden	Extended release tablets	Drug overdose	1	Nifedipine	Extended release tablets	Drug overdose
1	Oxycodone / Naloxone	Extended release tablets	Drug overdose	1	Trazodone	Extended release tablets	Drug overdose
1	Venlafaxine	Extended release tablets	Drug overdose				
